# National Ebola preparedness policy architecture and health system readiness in the Democratic Republic of the Congo and Uganda: a scoping review anchored in the 2026 Bundibugyo outbreak

**DOI:** 10.1186/s40249-026-01521-x

**Published:** 2026-11-05

**Authors:** Augustus Osborne, Morie Vandi, Hafsa Sentogo Lukwata, Camilla Bangura, Umar Sesay

**Affiliations:** 1 Institute for Development, Western Area, Freetown, Sierra Leone; 2 Ministry of Health, Youyi Building, Freetown, Sierra Leone; 3 Ministry of Health, Kampala, Uganda; 4 Department of Biological Sciences, School of Basic Sciences, Njala University, Private Mail Bag, Freetown, Sierra Leone; 5 Africa Centres for Disease Control and Prevention, Addis Ababa, Ethiopia

**Keywords:** Ebola, *Bundibugyo ebolavirus*, Preparedness policy, Health system, Cross-border health security, Scoping review, Democratic Republic of the Congo, Uganda

## Abstract

**Background:**

The 2026 Bundibugyo virus disease outbreak was declared in the Democratic Republic of the Congo (DRC) on 15 May 2026, and Uganda confirmed its first case the same day. Neither country had a licensed vaccine or treatment for the virus, yet the outbreak took very different courses in the two. This scoping review maps the evidence on their national preparedness instruments and health system readiness.

**Methods:**

We followed the Arksey and O'Malley framework, Joanna Briggs Institute guidance and the Preferred Reporting Items for Systematic reviews and Meta-Analyses extension for Scoping Reviews. National instruments in force at the onset, International Health Regulations evaluations and outbreak documents were sought from the issuing bodies, verified and charted against 52 component requirements in ten dimensions, with operational specificity as an eleventh. A fixed rule assigned each country a four-level descriptor per dimension, and documented outbreak risks were mapped against the gaps.

**Results:**

We included 57 sources, 15 of them national instruments. Uganda's instruments addressed 47 of 52 components (90.4%) and the DRC's 38 (73.1%), and 31.7% of Ugandan and 15.2% of Congolese provisions were highly specific. Uganda was rated higher in six dimensions, including surveillance, isolation capacity, displaced populations and accountability, and equal in five. Both were inadequate on cross-border governance, and neither had a Bundibugyo-specific countermeasure provision. In the 2023 Joint External Evaluations the mean indicator score was 2.75 in Uganda and 1.63 in the DRC (paired difference 1.13, 95% confidence interval 0.83 to 1.42). Uganda's outbreak ended with 20 confirmed cases, whereas the DRC had reported 7890 confirmed cases by 23 September. The DRC's response plan of May 2026 was highly specific in 21 of 37 provisions.

**Conclusions:**

The two countries differed in standing capacity and in the specificity of their standing instruments. The DRC's highly specific response plan suggests that its gap lay in standing arrangements rather than in response planning. A documentary review cannot establish that these differences caused the different courses of the outbreak. Priorities are surveillance and isolation capacity in the DRC, cross-border governance in both countries and national pathways for investigational Bundibugyo products.

**Graphical Abstract:**

**Figure:**
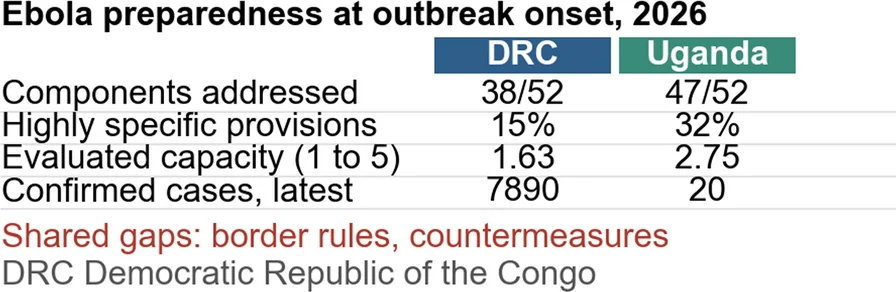

**Supplementary Information:**

The online version contains supplementary material available at https://doi.org/10.1186/s40249-026-01521-x.

## Background

**Fig. 1:**
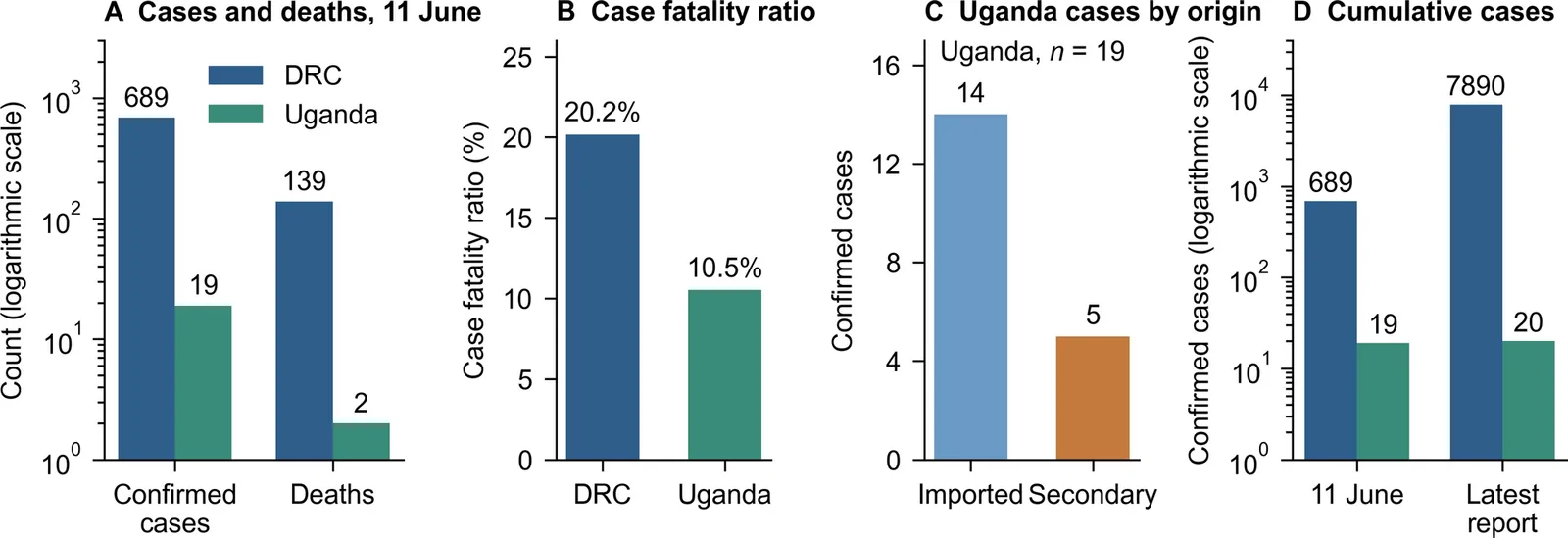
Epidemiological context of the 2026 Bundibugyo virus disease outbreak in the DRC and Uganda. **A** Confirmed cases and deaths to 11 June 2026 (logarithmic scale); **B** case fatality ratio among confirmed cases to 11 June 2026; **C** Ugandan confirmed cases by origin to 11 June 2026 (*n* = 19); **D** cumulative confirmed cases on 11 June 2026 and at the latest report (DRC, 23 September 2026; Uganda, end of outbreak, 27 August 2026; logarithmic scale). Source: prepared by the authors from the Africa Centres for Disease Control and Prevention situation report of 11 June 2026 [3], the DRC national situation report of 11 June 2026 [4], WHO Disease Outbreak News of 13 June and 25 September 2026 [5, 7] and the joint statement of 27 August 2026 [6]. DRC: Democratic Republic of the Congo, WHO: World Health Organization

The Ministry of Public Health, Hygiene and Social Welfare of the Democratic Republic of the Congo (DRC) declared an outbreak of Bundibugyo virus disease on 15 May 2026, and Uganda confirmed an imported case in Kampala on the same day [1]. The Director-General of the World Health Organization (WHO) determined on 17 May that the event was a public health emergency of international concern [2]. By 11 June the DRC had recorded 689 confirmed cases and 139 deaths in 29 health zones of Ituri, North Kivu and South Kivu, whereas Uganda had recorded 19 confirmed cases and 2 deaths, 14 of the cases imported (Fig. 1) [3–5]. The difference then widened. Uganda's outbreak was declared over on 27 August after 20 confirmed cases [6], while by 23 September the DRC had recorded 7890 confirmed cases and 3799 deaths in seven provinces [7].

Bundibugyo virus was first identified in Bundibugyo district, Uganda, in 2007 and caused a second outbreak in the DRC in 2012 [8, 9]. For Ebola virus (species *Zaire ebolavirus*), a vaccine and monoclonal antibody treatments have been shown to work in trials in Guinea and the DRC [10, 11]. For Bundibugyo virus no vaccine or treatment is licensed, and candidate products may be used only within clinical trials [12, 13]. Differences in access to licensed countermeasures therefore cannot explain why the outbreak took such different courses in the two countries.

The two countries reached 2026 by different routes. The 2018 to 2020 outbreak in North Kivu and Ituri, the largest in the DRC before 2026, caused 3481 cases and 2299 deaths, and its response was hampered by armed conflict, attacks on response teams and community distrust [14–16]. Uganda's 2022 Sudan virus disease outbreak caused 164 cases (142 confirmed and 22 probable) and 77 deaths; it was detected late but brought under control [17–19]. Displacement shapes risk on both sides of the border. In June 2026 more than 2 million forcibly displaced people lived in areas of the DRC at risk from the outbreak [20], and Uganda hosts more than 2 million refugees and asylum-seekers, about a third of them Congolese [21, 22]. During the West African epidemic, Ebola transmission in Montserrado County, Liberia, was greater in the poorest communities [23].

Published work on the two countries has examined single outbreaks and single responses, and the national preparedness instruments of the DRC and Uganda have not been compared against a common framework. This scoping review maps the evidence on national Ebola preparedness policy architecture and health system readiness in the two countries, using the 2026 outbreak as its anchor, and identifies gaps in policy and in the evidence base. The review questions are stated in full in the Methods.

## Methods

### Protocol and reporting framework

The review followed the framework of Arksey and O'Malley [24], the refinements proposed by Levac et al. [25] and the Joanna Briggs Institute (JBI) guidance for scoping reviews [26]. A scoping review was chosen because the aim was to map a heterogeneous body of policy, evaluation and outbreak documents and to identify gaps, not to estimate an effect [27]. Reporting follows the Preferred Reporting Items for Systematic reviews and Meta-Analyses (PRISMA) extension for Scoping Reviews (PRISMA-ScR) [28], and the completed checklist is in Additional file 1. The review was not registered; the review team agreed a protocol before the first search. Further methodological detail is in Additional file 2.

### Review questions

The questions were framed with the population, concept and context structure of the JBI guidance [26]. The population was the national health systems of the DRC and Uganda, including forcibly displaced people in both countries; the concept was national Ebola preparedness policy architecture and health system readiness; and the context was the onset of the 2026 Bundibugyo virus disease outbreak. The primary question was: what does the available policy, evaluation and outbreak evidence show about how the national Ebola preparedness instruments of the DRC and Uganda differ, and how do those differences relate to the course of the 2026 outbreak in each country? The three secondary questions were: in which governance and health system dimensions do the instruments of the two countries differ; which gaps in each country's instruments correspond to operational problems documented during the 2026 outbreak; and where is the evidence base itself thin? The review maps what documents state and what evaluations record. It does not assess implementation fidelity or clinical outcomes.

### Eligibility criteria

Eligible sources were national health sector plans, health security or multi-hazard emergency plans, Ebola-specific preparedness or response plans, public health legislation and emergency operations guidance of the DRC or Uganda; evaluations of either country's capacities under the International Health Regulations (IHR), including Joint External Evaluations (JEE), States Parties Self-Assessment Annual Reporting (SPAR) and after-action reviews; regional and continental instruments for cross-border epidemic preparedness covering the two countries; humanitarian documents on displaced populations and epidemic response in either country; peer-reviewed studies of Ebola preparedness, response or epidemic governance in either country; and situation reports, risk assessments and official statements on the 2026 outbreak. For each type of national instrument, the version in force on 15 May 2026 was charted. Where that version was not publicly available, its most recent public predecessor was charted and identified as such, and for Ebola-specific plans the most recent national plan issued before the onset was charted. Instruments issued after the onset were charted as response-phase instruments and were not used to assign descriptors. Sources were excluded if they dealt only with clinical management or virology, were subnational plans without a stated issuing body, or could not be traced to a published document. English and French sources were eligible.

### Information sources and search

The first search, in June 2026, covered PubMed (https://pubmed.ncbi.nlm.nih.gov), Global Health on the platform of the Centre for Agriculture and Bioscience International (https://www.cabidigitallibrary.org), the WHO Institutional Repository for Information Sharing (https://iris.who.int), the publications of the Africa Centres for Disease Control and Prevention (Africa CDC, https://africacdc.org) and of the East African Community (https://www.eac.int), the data portal of the United Nations High Commissioner for Refugees (UNHCR, https://data.unhcr.org) and the websites of the two ministries of health. In September 2026 a targeted search was made for Ugandan public health legislation, Uganda's national action plan for health security and UNHCR data for Uganda. The search strategies and the screening yield of the June search are in Additional file 3. At revision, on 1 October 2026, the national instruments in force at the onset of the outbreak, the JEE mission reports, SPAR data and the outbreak documentation to that date were sought directly from the issuing bodies: the Institut National de Santé Publique (INSP) of the DRC (https://insp.cd), the Uganda Ministry of Health (https://www.health.go.ug), the WHO Strategic Partnership for Health Security portal (https://extranet.who.int/sph), the WHO Global Health Observatory (https://www.who.int/data/gho), WHO Disease Outbreak News, the WHO Regional Office for Africa (https://www.afro.who.int), Africa CDC, UNHCR and the Uganda Legal Information Institute (https://ulii.org). Where the issuing body's copy of an instrument could not be found or opened, another copy was read, and Additional file 4 identifies it.

### Verification of sources at revision

At revision every source included in the first version of the review, and every statement of fact drawn from it, was checked against the issuing body's own record or against PubMed. Citations with wrong details were corrected to the document actually issued. Sources that could not be traced to any published document were removed, and the analysis that had rested on them was redone from the instruments themselves, sought from the issuing bodies. Traceable sources that did not meet the eligibility criteria, or that were superseded by documents closer to the data cut-off, were removed from the included set and, where relevant, kept as background references. The outcome for each source is recorded in Additional file 4.

### Selection of sources of evidence

**Fig. 2:**
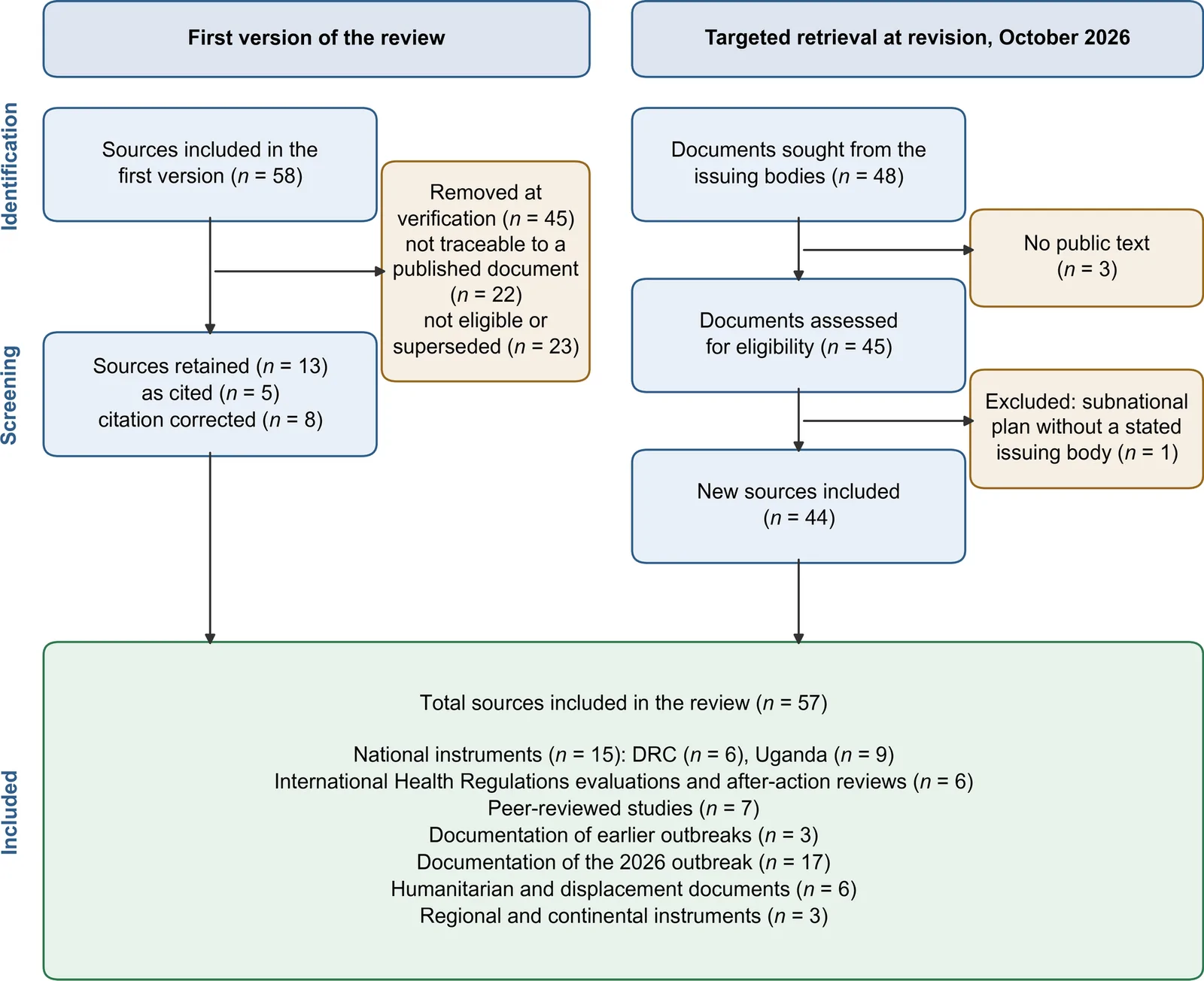
Flow diagram of the selection of sources of evidence, adapted from the PRISMA 2020 flow diagram for updated reviews [29]. The left column shows the verification at revision of the 58 sources included in the first version of the review; the right column shows the documents sought from the issuing bodies at revision. The screening of the June 2026 search is reported in Additional file 3. DRC: Democratic Republic of the Congo, PRISMA: Preferred Reporting Items for Systematic reviews and Meta-Analyses

Two reviewers screened titles and abstracts, or document metadata for grey literature, and assessed full texts against the eligibility criteria, and disagreements were resolved with a third reviewer. Selection is reported in a flow diagram adapted from the PRISMA 2020 diagram for updated reviews [29] (Fig. 2).

### Source credibility

Formal critical appraisal is optional in scoping reviews [26, 28]. Because most sources were policy or grey-literature documents, each was rated for provenance, official or peer-reviewed status, currency relative to the outbreak and completeness of relevant content. The ratings are in Additional file 4 and were not used to exclude sources.

### Data charting

The charting framework has eleven dimensions: preparedness governance; surveillance; inclusion of forcibly displaced populations; isolation and treatment capacity; health workforce readiness; cross-border governance; community engagement and risk communication; access to medical countermeasures; accountability and monitoring; alignment with WHO and Africa CDC standards; and operational specificity. The first ten comprise 52 component requirements drawn from the WHO consolidated Ebola virus disease preparedness checklist [30] and the third edition of the JEE tool [31]; those for displaced populations were drawn from work on the inclusion of refugees in national public health systems and on public health information in crises [32, 33]. Each area of the joint Africa CDC and WHO continental preparedness and response plan for the 2026 outbreak, from coordination and surveillance to cross-border collaboration and the protection of vulnerable populations, corresponds to one or more of these dimensions [34]. The components and their provenance are in Additional file 5. Each national instrument was searched for a provision addressing each component. All were read in full except for the costing slide and some annex tables of the DRC development plan, which were illegible in the only copy available, image-only project profiles in an annex of Uganda's strategic plan II, and parts of Uganda's Public Health Act and Constitution. What was read of each instrument is recorded in Additional file 4. For each provision we recorded a summary, a verbatim quotation with its page, the institution named, any timeline, any quantified target or resource, the level of the health system and its operational specificity: high (concrete actions, named institutions, timelines and quantified resources), moderate (procedural guidance with some institutional attribution but little quantification) or low (aspirational or generic language). French instruments were charted from the French text and are quoted in French. The charted data are in Additional file 4.

### Synthesis and analysis

For each country and dimension, two properties of the charted provisions were combined by a rule fixed before synthesis: coverage (full when every component requirement was addressed, partial when some were and none when none were) and the modal specificity of the provisions. Full coverage with high modal specificity was rated adequate; full coverage with moderate, or partial coverage with high or moderate, specificity was rated partially adequate; low modal specificity was rated inadequate; and no coverage was rated absent. A tie in specificity was resolved upwards. For the operational specificity dimension, the descriptor is the modal specificity of all provisions. The rule and the reading for every country and dimension are in Additional file 6. Counts of sources, components and provisions are reported as numbers and percentages. No inferential test was applied to the charted provisions, which are a census of the provisions in the instruments rather than a sample. JEE indicator scores (1 to 5) were extracted from the 2023 mission reports and summarised as means and as counts by level. The paired difference across the 56 indicators is reported with a 95% confidence interval and a Wilcoxon signed-rank test, used to describe how consistent the difference was across indicators rather than to generalise beyond them. SPAR average capacity scores for 2018 to 2025 were taken from the WHO Global Health Observatory [35].

### Gap analysis and outbreak-response risk

Policy gaps were identified from the descriptors. For eight gap dimensions, the outbreak documentation up to 20 June 2026 was then read for the operational consequences of each gap, and a four-level risk descriptor was assigned: critical (a documented consequence impeding detection, isolation or treatment at scale, or no mitigating provision anywhere in the national or regional architecture), significant (an adverse consequence confined to part of the affected area, or partly mitigated), moderate (a limitation of function without a documented adverse outcome) or low (no gap of operational consequence documented). Definitions and the documentary basis of each descriptor are in Additional file 6. An evidence-gap analysis identified where the charted evidence was thin or provisional.

### Software

Counts, percentages, descriptors, the paired comparison and the figures were produced in Python 3.11 (Python Software Foundation, Wilmington, Delaware, USA; https://www.python.org) with the SciPy, matplotlib and openpyxl libraries. The charting workbook (Additional file 4) recalculates the counts, percentages, descriptors and confidence interval by formula; its formulas were evaluated in LibreOffice Calc 24.2 (The Document Foundation, Berlin, Germany; https://www.libreoffice.org).

## Results

### Selection of sources of evidence

The first version of the review included 58 sources (Fig. 2). At verification, 22 could not be traced to any published document and were removed. A further 23 were traceable but were removed from the included set: 22 did not meet the eligibility criteria, most of them virological, clinical or general studies, and 1 situation report was superseded by reports closer to the data cut-off. Several are now cited as background. Thirteen were retained, 8 of them with corrected citation details. At revision 48 documents were sought directly from the issuing bodies. Three national instruments had no public text. For Uganda's second national action plan for health security (2024 to 2029) and its health sector refugee response plan for 2025–2029, their predecessors were charted; for the DRC's national action plan for health security, cited by its multi-hazard plan, no version could be found [36]. Of the 45 documents assessed, 1 subnational plan without a stated issuing body was excluded and 44 were included; no national Ebola-specific plan issued by the DRC before the onset was publicly available. The review therefore includes 57 sources (*n* = 57), which are listed with their verification status in Additional file 4.

### Characteristics of sources of evidence

Table 1 summarises the 57 sources. Fifteen (26.3%) were national instruments—6 for the DRC and 9 for Uganda—and 17 (29.8%) documented the 2026 outbreak. Forty-three (75.4%) were issued in 2023 or later, and 9 (15.8%) were in French, all of which were Congolese. Provenance was rated moderate for 7 sources (12.3%): 5 national instruments read from uncertified copies hosted outside the issuing body's website, a press release and a news report. Every count in this section can be traced to Additional file 4.

**Table 1:** Characteristics of the included sources of evidence (*n* = 57)

Characteristic	DRC (*n* = 18)	Uganda (*n* = 23)	Both countries or regional (*n* = 16)	Total (*n* = 57)
Source type				
National instrument	6 (33.3)	9 (39.1)	0 (0.0)	15 (26.3)
IHR evaluation or after-action review	2 (11.1)	3 (13.0)	1 (6.3)	6 (10.5)
Peer-reviewed study	3 (16.7)	4 (17.4)	0 (0.0)	7 (12.3)
Documentation of an earlier outbreak	0 (0.0)	3 (13.0)	0 (0.0)	3 (5.3)
Documentation of the 2026 outbreak	5 (27.8)	2 (8.7)	10 (62.5)	17 (29.8)
Humanitarian or displacement document	2 (11.1)	2 (8.7)	2 (12.5)	6 (10.5)
Regional or continental instrument	0 (0.0)	0 (0.0)	3 (18.8)	3 (5.3)
Year of issue				
2019 or earlier	5 (27.8)	4 (17.4)	0 (0.0)	9 (15.8)
2020 to 2022	1 (5.6)	4 (17.4)	0 (0.0)	5 (8.8)
2023 to 2025	4 (22.2)	11 (47.8)	0 (0.0)	15 (26.3)
2026	8 (44.4)	4 (17.4)	16 (100.0)	28 (49.1)
Language				
English	9 (50.0)	23 (100.0)	16 (100.0)	48 (84.2)
French	9 (50.0)	0 (0.0)	0 (0.0)	9 (15.8)
Status				
Official document	13 (72.2)	19 (82.6)	15 (93.8)	47 (82.5)
Peer-reviewed article	3 (16.7)	4 (17.4)	0 (0.0)	7 (12.3)
Other (press release, news report or editorial)	2 (11.1)	0 (0.0)	1 (6.3)	3 (5.3)
Provenance				
High	13 (72.2)	21 (91.3)	16 (100.0)	50 (87.7)
Moderate	5 (27.8)	2 (8.7)	0 (0.0)	7 (12.3)
Currency relative to the outbreak				
High	13 (72.2)	10 (43.5)	16 (100.0)	39 (68.4)
Moderate	3 (16.7)	11 (47.8)	0 (0.0)	14 (24.6)
Low	2 (11.1)	2 (8.7)	0 (0.0)	4 (7.0)
Completeness of relevant content				
High	5 (27.8)	9 (39.1)	2 (12.5)	16 (28.1)
Moderate	11 (61.1)	12 (52.2)	13 (81.3)	36 (63.2)
Low	2 (11.1)	2 (8.7)	1 (6.3)	5 (8.8)
Verification status				
Retained from the first version as cited	0 (0.0)	5 (21.7)	0 (0.0)	5 (8.8)
Retained with corrected citation	2 (11.1)	3 (13.0)	3 (18.8)	8 (14.0)
Retrieved at revision	16 (88.9)	15 (65.2)	13 (81.3)	44 (77.2)

Values are *n *(%) of the column total. Currency was rated relative to the onset of the outbreak on 15 May 2026. DRC: Democratic Republic of the Congo, IHR: International Health Regulations

### Charted provisions and descriptors across the eleven dimensions

**Fig. 3:**
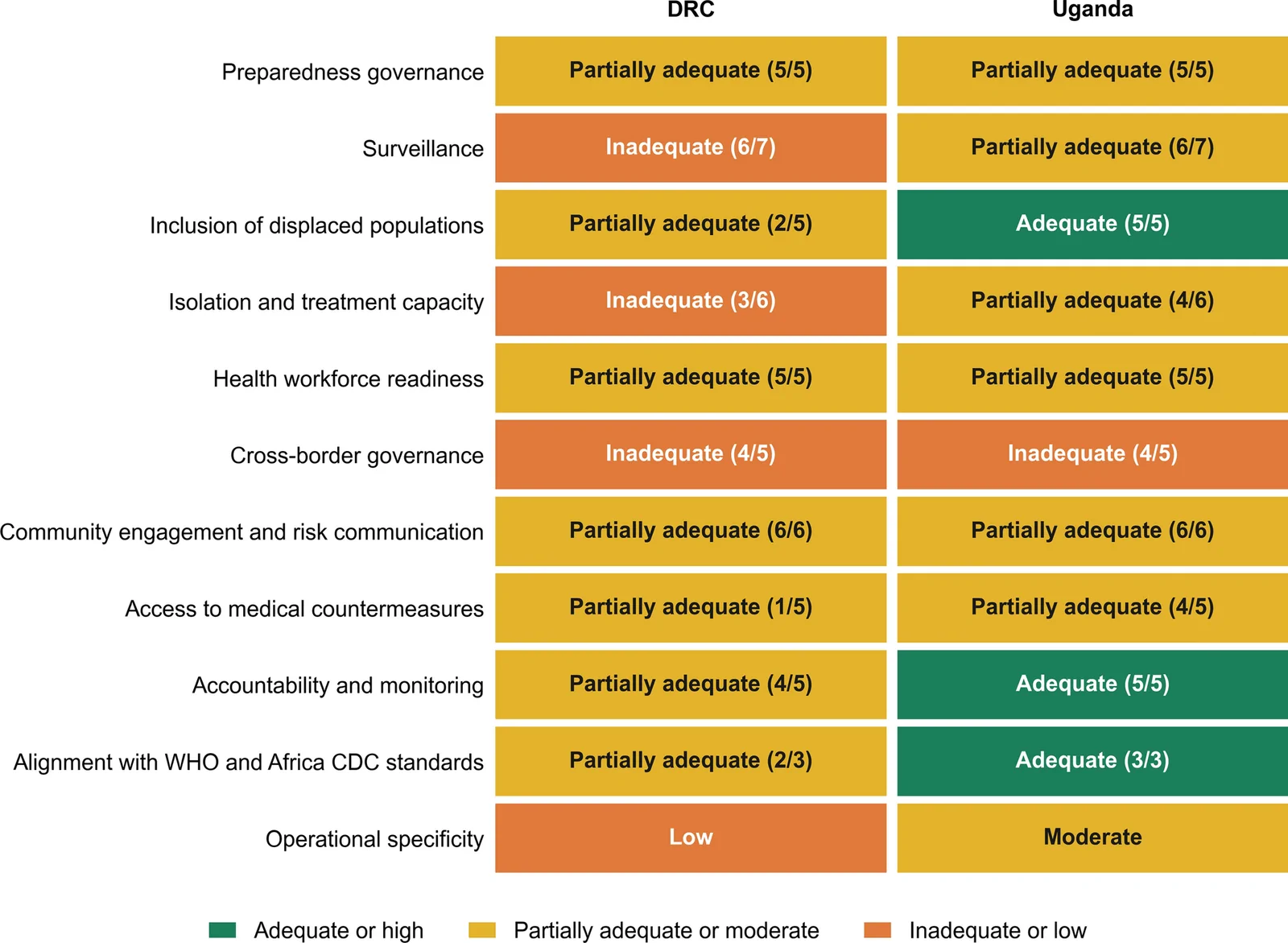
Descriptors of the national instruments of the DRC and Uganda in force at the onset of the 2026 outbreak, by dimension, corresponding to Table 2. Each cell gives the descriptor and the number of component requirements addressed. Source: prepared by the authors from the charting in Additional file 4. DRC: Democratic Republic of the Congo, WHO: World Health Organization, Africa CDC: Africa Centres for Disease Control and Prevention

Charting of the instruments in force at the onset produced 66 provisions for the DRC and 142 for Uganda (Table 2). Uganda's instruments addressed 47 of the 52 component requirements (90.4%) and the DRC's 38 (73.1%). Highly specific provisions made up 31.7% of Ugandan provisions (45 of 142) and 15.2% of Congolese provisions (10 of 66), and provisions of low specificity 15.5% (22 of 142) and 45.5% (30 of 66). Uganda was rated higher than the DRC in six of the eleven dimensions (surveillance, inclusion of displaced populations, isolation and treatment capacity, accountability, alignment with standards and operational specificity) and the same in five (governance, workforce, cross-border governance, community engagement and access to countermeasures). The DRC was not rated higher in any dimension, and no dimension was rated absent for either country (Table 2; Fig. 3). Resolving the three ties in specificity downwards rather than upwards would change three descriptors but would still leave Uganda rated higher in six dimensions (Additional file 6).

**Table 2:** Coverage, operational specificity and descriptor of the national instruments in force at the onset of the 2026 outbreak, by country and dimension

Dimension	DRC coverage, *n*/*N*	DRC provisions (high, moderate, low)	DRC descriptor	Uganda coverage, *n*/*N*	Uganda provisions (high, moderate, low)	Uganda descriptor
Preparedness governance	5/5	1/6/3	Partially adequate	5/5	4/15/2	Partially adequate
Surveillance	6/7	2/3/8	Inadequate	6/7	12/15/2	Partially adequate
Inclusion of displaced populations	2/5	0/1/1	Partially adequate	5/5	5/1/1	Adequate
Isolation and treatment capacity	3/6	1/1/4	Inadequate	4/6	2/8/2	Partially adequate
Health workforce readiness	5/5	2/4/4	Partially adequate	5/5	4/11/4	Partially adequate
Cross-border governance	4/5	0/1/4	Inadequate	4/5	2/2/3	Inadequate
Community engagement and risk communication	6/6	1/4/3	Partially adequate	6/6	2/12/3	Partially adequate
Access to medical countermeasures	1/5	1/0/0	Partially adequate	4/5	1/4/1	Partially adequate
Accountability and monitoring	4/5	2/3/2	Partially adequate	5/5	10/4/3	Adequate
Alignment with WHO and Africa CDC standards	2/3	0/3/1	Partially adequate	3/3	3/3/1	Adequate
Operational specificity (all provisions)		10/26/30	Low		45/75/22	Moderate
Total	38/52 (73.1%)	10/26/30 (66)		47/52 (90.4%)	45/75/22 (142)

Coverage, component requirements addressed by at least one provision, *n*/*N*; provisions, number of charted provisions rated high, moderate and low, separated by slashes. Descriptor rule (Additional file 6): full coverage with high modal specificity, adequate; full coverage with moderate, or partial coverage with high or moderate, specificity, partially adequate; low modal specificity, inadequate; no coverage, absent. For operational specificity the descriptor is the modal specificity of all provisions. DRC instruments: national multi-hazard plan for public health emergencies 2024 to 2027, national health development plan 2024 to 2033 and public health law no. 18/035 as amended in 2023 [36–39]. Uganda instruments: Ministry of Health strategic plan II 2025/26 to 2029/30, national action plan for health security 2019 to 2023, health sector integrated refugee response plan 2019 to 2024, national Ebola response plan 2022, guidelines for regional public health emergency operations centres 2023, Public Health Act and its 2023 amendment, and the Constitution [40–47]. DRC Democratic Republic of the Congo, WHO World Health Organization, Africa CDC Africa Centres for Disease Control and Prevention.

#### Governance and legal basis

Both countries' instruments covered all five governance components, and both were rated partially adequate because most governance provisions were moderately specific. In the DRC, the national multi-hazard plan places a coordination council for epidemics, emergencies and complex disasters under the Prime Minister, alongside a task force led by the Minister of Health and the public health emergency operations centre (COUSP) of the INSP, and sets out how the Minister's declaration activates the centre and its incident management system [36]. The public health law as amended in 2023 creates the coordination council and charges the INSP with applying the IHR, but gives no authority the power to declare an epidemic, to order isolation or quarantine, or to restrict movement; control measures are left to ministerial order [37, 38]. The 2023 JEE recorded that no legal text established the COUSP or regulated quarantine, and that the three Ebola outbreaks preceding the evaluation had been managed by a technical secretariat set up by presidential order [48]. In Uganda, the Public Health Act empowers the Minister to declare a disease epidemic, to declare infected areas and to make rules restricting movement out of them, and the Constitution provides for a state of emergency, although it does not name epidemics [40–42]. National guidelines set activation criteria, modes of operation and a notification chain for regional emergency operations centres [43], and the 2023 JEE recorded a national emergency operations centre with regional centres while noting that IHR coordination was not yet institutionalised [49].

#### Surveillance, isolation and workforce

Surveillance showed the widest difference in specificity. Uganda's instruments addressed six of the seven surveillance components, and 12 of its 29 surveillance provisions were highly specific. Its action plan for health security sets training in integrated disease surveillance and response year by year, names seven national laboratories and plans specimen transport from at least 80% of districts [44]; the strategic plan in force at the onset sets targets for detecting emergencies within 72 h, confirming outbreaks within 7 days and raising the number of functional points of entry from 8 to 17 [45]; and the regional guidelines require daily reporting and use the 7-1-7 timeliness target [43]. The DRC also addressed six of the seven components, but 8 of its 13 surveillance provisions were of low specificity. Its multi-hazard plan sets a laboratory network and a 48-h detection target, yet its Ebola provisions state no case definition and no timeliness standard for electronic reporting [36]. Neither country's standing instruments provide for surveillance in insecure or inaccessible areas.

For isolation and treatment, Uganda's instruments addressed four of the six components and the DRC's three. Neither country sets a minimum bed provision or surge triggers in a standing instrument, and the only named treatment facility is the Mubende unit in Uganda's 2022 Ebola response plan [46]. The DRC multi-hazard plan lists case-management kits for 500 patients as required and none as available [36], whereas Uganda's provisions concern supplies pre-positioned at regional referral hospitals, conveyance of patients and ambulance services [40, 44, 45]. Workforce provisions covered all five components in both countries, and both were rated partially adequate. The DRC plan lists 188 rapid response teams and 1000 personal protective equipment kits as required and none of either as available [36]. Uganda's provisions specify the cadres to be trained and the content of training, and twice-yearly exercises for national, regional and district rapid response teams, but set no stockpile levels [43, 44].

#### Inclusion of displaced populations

Uganda's health sector integrated refugee response plan for 2019 to 2024 addressed all five components of this dimension, most of them with high specificity. It harmonises the district and refugee health information systems under Ministry of Health authority, recruits and pays community health workers in refugee-hosting districts, places the response under a steering committee co-chaired by the Ministry and including the Office of the Prime Minister and UNHCR, and moves settlement health services into the district health system [47]. Its epidemic provisions are general: registers for community-based disease surveillance, harmonised disease reporting, standby emergency health teams, a 48-h detection target and the screening of new arrivals. The strategic plan in force at the onset commits the Ministry to integrating refugee health into government services, and the plan's successor for 2025 to 2029 has been announced but not published [45, 50]. Uganda's 2023 JEE report does not mention refugees [49], and its 2022 Ebola response plan refers to refugee settlements only for the activation of response teams, materials in refugee languages and a survey of knowledge, attitudes and practices [46]. In the DRC, two of the five components were addressed. The multi-hazard plan treats displacement as a separate hazard. It assigns displacement to the Ministry of the Interior and sets continuity of primary care for displaced people within 48 h, without reference to humanitarian health providers or to Ebola [36].

#### Cross-border governance and community engagement

Cross-border governance was rated inadequate in both countries. The instruments of both touch on information exchange and the control of movement, but none sets a bilateral notification obligation with a timeline, or criteria for restricting movement with humanitarian mitigation. Uganda's Public Health Act empowers the Minister to restrict entry from specified countries without stating criteria [40], and the DRC plan assigns cross-border information exchange to Interpol and control of population movement to the migration authority [36]. Uganda's 2022 Ebola plan provides for cross-border meetings with Congolese health officials [46]. A bilateral memorandum of understanding between the two countries was launched in Aru on 23 June 2026, after the onset [51]. Community engagement covered all six components in both countries and was rated partially adequate in both. The DRC multi-hazard plan names the national programme responsible for risk communication, differentiates audiences, provides for rumour management and sets safe and dignified burial within 24 h in one section and 48 h in another [36]; Uganda assigns risk communication on emergencies to the Ministry's health promotion department and provides for burial teams in each high-risk district [45, 46].

#### Countermeasures, accountability and alignment with standards

Neither country had a provision for a Bundibugyo-specific vaccine or treatment. The only Congolese provision charted under countermeasures is the yearly pre-positioning of response vaccines, with an Ebola scenario requiring 24,000 vaccine doses and two antibody treatments, all of them products for Ebola virus (*Zaire ebolavirus*), listed as required and not available [36]. The public health law also allows the Minister to order compulsory vaccination when an epidemic appears, without reference to any product [37]. Uganda's 2022 Ebola plan recorded that the available vaccine was specific to *Z. ebolavirus* and set out the approvals needed for investigational products from an institutional review board, the Uganda National Council for Science and Technology and the National Drug Authority, under what it called a compassionate approach [46]. That route was used in 2022, when candidate Sudan virus vaccines arrived 79 days after the outbreak was declared, and in 2025, when a ring vaccination trial began four days after a new Sudan virus outbreak was confirmed [52, 53].

Accountability was rated adequate in Uganda and partially adequate in the DRC. Uganda's action plan for health security requires after-action reviews of every activation of the emergency operations centre and an annual review that uses the WHO benchmarks, SPAR, simulation exercises and after-action reviews to set the next year's priorities [44], and a national after-action review of the 2022 response was held in February 2023 [54]. The same plan is the only instrument in either country that reports its own provisions against each JEE indicator, which, with a tie between high and moderate provisions resolved upwards, gives Uganda its adequate rating on alignment with standards [44]. The DRC's development plan sets emergency indicators with baselines and annual targets [39], but no Congolese standing instrument requires after-action review or maps its provisions against the JEE.

#### The response plan issued after the outbreak was declared

By 29 May 2026 the INSP had issued a One Health preparedness and response plan for the outbreak in Ituri and North Kivu, coordinated through the COUSP and its incident management system [55]. Charted separately as a response-phase instrument, it addressed 37 of the 52 components, and 21 of its 37 provisions (56.8%) were highly specific, against 10 of 66 (15.2%) in the standing Congolese instruments. It sets four planning scenarios by number of cases, a 12-h target for laboratory confirmation, daily situation reports, protection of response teams in insecure areas, ambulances for every affected health zone and regular payment of frontline allowances [55]. It also lists products for *Z. ebolavirus* in its research section, and its text and budget give different numbers of points of entry. No equivalent Ugandan plan for the 2026 outbreak was publicly available.

### Policy gaps mapped against outbreak-response risks

**Fig. 4:**
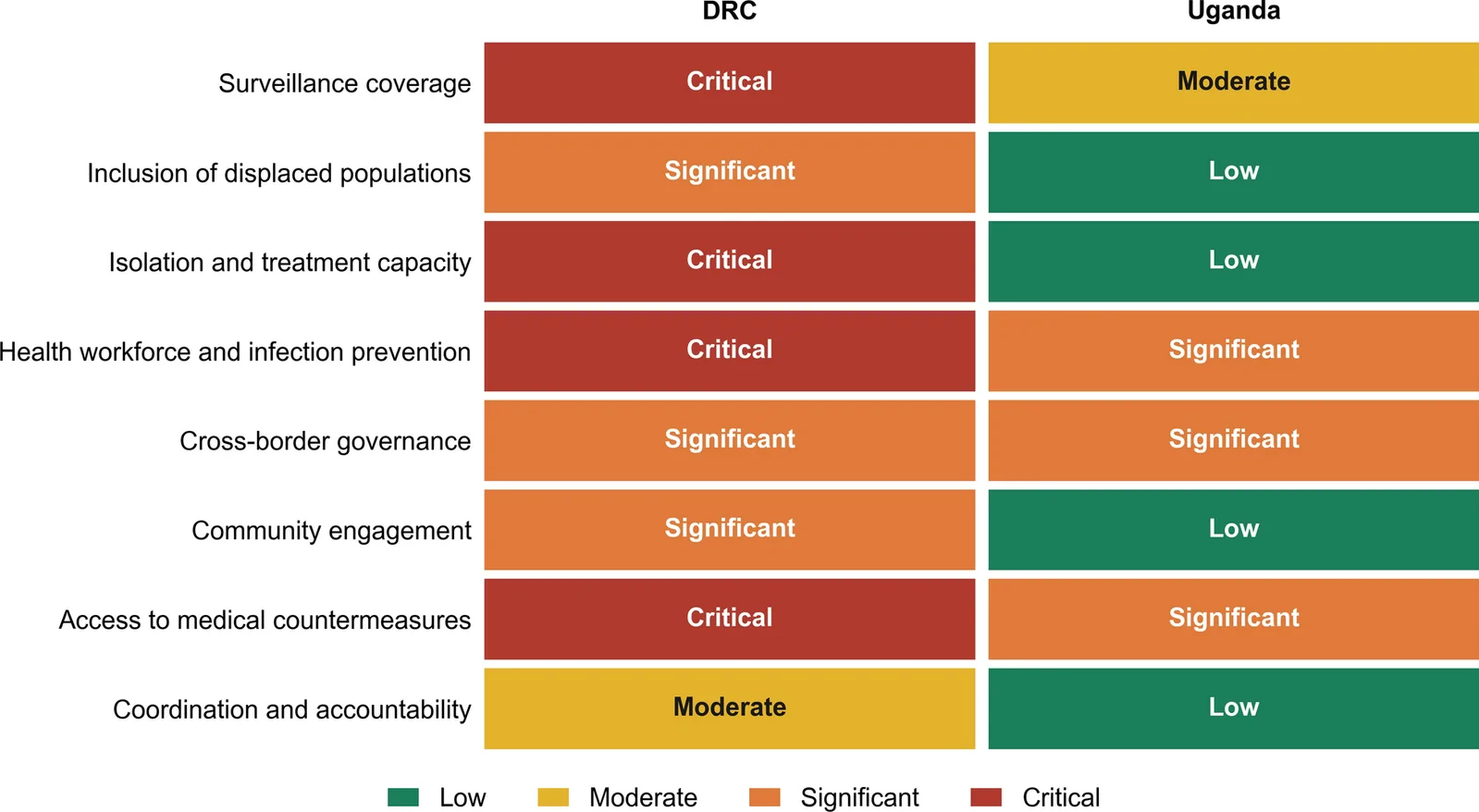
Policy gaps mapped against the outbreak-response risks documented in the DRC and Uganda up to 20 June 2026, corresponding to Table 3. Source: prepared by the authors from the charting in Additional file 4 and the outbreak documentation cited in Table 3. DRC: Democratic Republic of the Congo

Table 3 and Fig. 4 set the policy gaps against the risks documented up to 20 June 2026. Four risks were rated critical in the DRC and none in Uganda. In the DRC, WHO reported surveillance disrupted by insecurity and suspected cases traced back to March, and the national report of 11 June recorded that 28.4% of 6043 contacts had been seen, 17.4% in Ituri, although WHO's figures for the previous day were higher [4, 5, 56]. Isolation beds in Ituri were 77.9% occupied and treatment centres there were described as overwhelmed [4, 57], 16 health workers had been infected [5], and no countermeasure trial had started [5]. In Uganda, the first case was confirmed only after death, and four health workers were infected while attempting to resuscitate a probable case [1, 58]. Uganda suspended passenger transport across the border, a measure contrary to the IHR temporary recommendations and to Africa CDC guidance, but its confirmed cases rose from 9 on 29 May to 19 by 11 June, 14 of them imported [5, 56, 59–61]. Displacement-related risk was significant in the DRC, where displaced people blocked response teams after two Ebola deaths at the Kpangba site near Bunia, and low in Uganda, where no refugee was among the cases [20].

**Table 3:** Policy gaps mapped against the outbreak-response risks documented in the DRC and Uganda up to 20 June 2026

Gap dimension	DRC: documented risk	Uganda: documented risk
Surveillance coverage	Critical. Surveillance disrupted by insecurity; suspected cases traced back to March; transmission probably undetected for weeks in newly reporting zones; 28.4% of 6043 contacts seen on 11 June in the national report (17.4% in Ituri), 71% to 84% by province in WHO data for 10 June [4, 5, 56, 62]	Moderate. First case confirmed on 15 May, the day after death; no documented community transmission; 394 of 820 contacts had completed follow-up by 11 June [1, 5]
Inclusion of displaced populations	Significant. Two Ebola deaths at the Kpangba displacement site near Bunia; response teams blocked on 3 June; WHO warned of undetected transmission among mobile and displaced people [5, 20]	Low. No refugee among the 19 confirmed cases [20]
Isolation and treatment capacity	Critical. 452 isolation beds 69.7% occupied on 11 June (77.9% in Ituri, with 646 of 689 cases); capacity reported as inadequate and Ituri treatment centres as overwhelmed [4, 56, 57]	Low. Patients isolated in designated centres, including Mulago; no shortfall reported [3, 58]
Health workforce and infection prevention	Critical. 16 health workers infected by 10 June, four of whom died within four days at one hospital; stock-outs of protective and infection prevention supplies [5, 56, 63]	Significant. Four health workers infected while resuscitating a probable case who was embalmed before testing [58]
Cross-border governance	Significant. 14 of Uganda's 19 cases imported by 11 June; no bilateral protocol before the memorandum of 23 June [5, 51]	Significant. Passenger transport suspended, contrary to IHR temporary recommendations; importations continued; informal crossings expected to persist [5, 56, 59]
Community engagement	Significant. Security incidents and community resistance in Mongbwalu and Rwampara health zones; poor acceptance of safe burial; refusal of post-mortem sampling [4, 56, 61]	Low. No community resistance reported [5]
Access to medical countermeasures	Critical. No licensed product; trial protocols still under ethics and regulatory review on 13 June; no national access route in standing instruments [5, 12]	Significant. No licensed product; all 18 patients treated at Mulago received remdesivir under compassionate use [58]
Coordination and accountability	Moderate. Coordination limited in remote and insecure areas despite national and provincial activation [3, 56]	Low. No coordination failure reported [3, 5]

Risk descriptors (critical, significant, moderate, low) are defined, with their documentary basis, in Additional file 6. DRC Democratic Republic of the Congo, IHR International Health Regulations, WHO World Health Organization.

### Externally assessed capacity and preparedness trajectories

**Fig. 5:**
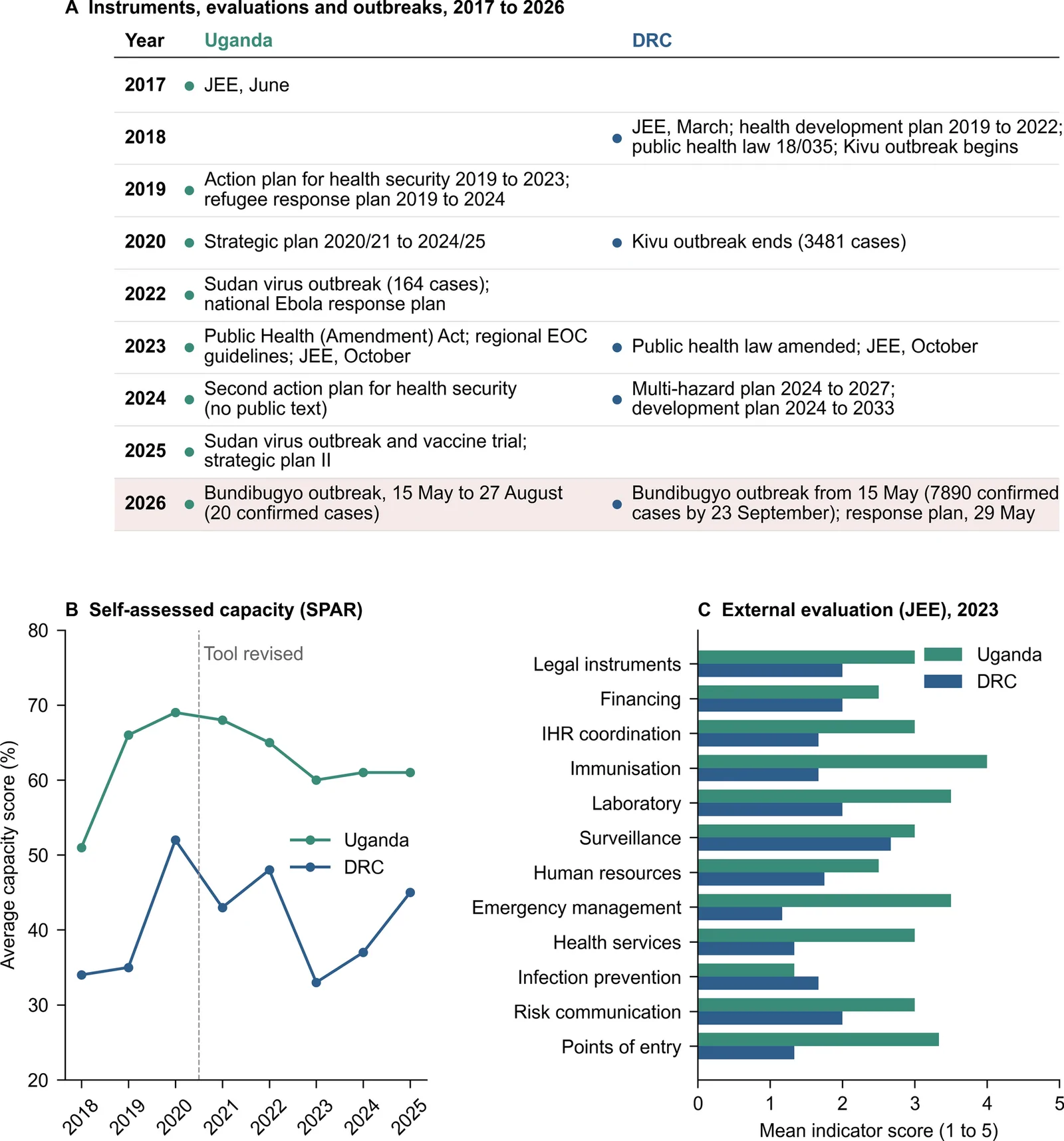
Preparedness trajectories of the DRC and Uganda. **A**, National instruments charted in this review, JEE missions and Ebola disease outbreaks, 2017 to 2026; **B**, SPAR average capacity score, 2018 to 2025 (13 capacities to 2020 and 15 from 2021); **C**, mean JEE indicator score in 12 of the 19 technical areas, October 2023 missions (all areas are in Additional file 4). Source: prepared by the authors from the instruments and evaluations charted in this review [48, 49, 64, 65, 69, 70], the outbreak documentation cited in the text [6, 7, 14, 17, 53] and WHO Global Health Observatory data [35]. DRC: Democratic Republic of the Congo, EOC: emergency operations centre, IHR: International Health Regulations, JEE: Joint External Evaluation, SPAR: States Parties Self-Assessment Annual Reporting, WHO: World Health Organization

The JEE missions of October 2023, which used the same 56-indicator tool in both countries, gave a mean indicator score of 2.75 for Uganda and 1.63 for the DRC [48, 49]. Uganda scored higher on 40 indicators (71.4%), the same on 13 (23.2%) and lower on 3 (5.4%), a mean paired difference of 1.13 points (95% confidence interval 0.83 to 1.42; Wilcoxon signed-rank *P* < 0.001). Twelve Ugandan indicators and no Congolese indicator reached level 4 or 5, whereas 29 Congolese indicators (51.8%) were at level 1. The largest differences were in health emergency management (mean 3.50 against 1.17) and immunisation (4.00 against 1.67), followed by points of entry (3.33 against 1.33); infection prevention and control was the one technical area in which Uganda scored lower (1.33 against 1.67). The first JEEs, in 2017 in Uganda and 2018 in the DRC, used an earlier edition of the tool and gave means of 2.70 and 1.85 [64, 65]. SPAR average capacity scores, which are self-reported [66], were higher in Uganda in every year from 2018 to 2025, by 16 to 31 percentage points (means 62.6% and 40.9%) [35] (Fig. 5). Uganda had activated task forces and response teams in 30 high-risk districts during the Kivu outbreak of 2018 and 2019 [67], and evaluations of its 2022 response reported delayed detection and barriers to community engagement as well as eventual control [18, 19, 68]. All the standing plans and guidelines charted for both countries were adopted between 2019 and 2025, so the difference in their specificity was in place before the outbreak began.

### Gaps in the evidence base

Four gaps in the evidence base stand out. First, the national instruments in force at the onset were not all public: Uganda's second action plan for health security and its current refugee response plan could not be retrieved, nor could any version of the DRC's action plan for health security, and five charted instruments were read from uncertified copies hosted outside the issuing body's website. Second, the evidence documents what instruments say rather than what was done, and no source evaluated the implementation of the charted provisions in either country. Third, the outbreak evidence comes from reports issued during an active outbreak, and figures for the same date differed between sources, as with contact follow-up in Ituri. Fourth, peer-reviewed evidence on epidemic governance in displacement settings and on the working of cross-border mechanisms between the two countries is sparse; the bilateral memorandum of June 2026 has been described but not evaluated.

## Discussion

This review compared the national preparedness instruments of the DRC and Uganda that were in force when the 2026 Bundibugyo virus disease outbreak began, and set them against externally assessed capacity and against the risks documented during the outbreak. Uganda's instruments were more complete and more specific, Uganda was rated higher in six of eleven dimensions and lower in none, and its externally assessed IHR capacity was higher on most indicators. The outbreak was contained in Uganda and spread widely in the DRC. These findings are consistent with preparedness architecture contributing to the different courses of the outbreak, but they do not show that it was the deciding factor.

The contrast between the DRC's standing instruments and its response plan is perhaps the most informative finding. Fewer than one in six provisions in the standing Congolese instruments were highly specific, but more than half of those in the plan issued within two weeks of the declaration were. The DRC was therefore able to write an operationally specific plan once an outbreak had been declared. What the standing instruments did not provide, and what the 2023 JEE also recorded as weak, were the arrangements that have to be in place before an outbreak: a legal basis for the emergency operations centre and for quarantine, health emergency management, surveillance with defined timeliness and the teams and stocks that the multi-hazard plan lists as required and records as unavailable [36, 48]. Uganda's instruments describe many of these standing arrangements, and its JEE scores for health emergency management and points of entry were more than twice those of the DRC. A response plan written after a declaration is unlikely to supply in two weeks what a standing system builds over years, although this review could not test that directly.

The finding on displaced populations runs against what the scale of displacement in the DRC, more than 6 million internally displaced people in June 2026 [71], might suggest. Uganda's refugee response plan was the only instrument in either country to address all five components, and its approach of moving settlement health services into the district system matches the case made for including refugees in national public health systems [32]. The Congolese instruments treat displacement as a separate hazard and do not connect it to epidemic surveillance or to the humanitarian providers who deliver much of the care in displacement sites. During the outbreak, problems arose in this area in Ituri, where displaced people blocked response teams and cases later reached displacement camps [20, 72], although the documents cannot show whether the gap in the instruments contributed to them. Public health information in crisis-affected populations is generally weak [33], which makes the absence of any link between national surveillance and humanitarian health information in the Congolese instruments the more notable. Uganda's plan, however, has only general epidemic provisions, and its successor has not been published.

Cross-border governance was weak in both countries, and the outbreak tested it. Neither country's instruments set notification timelines or criteria for restricting movement, and Uganda's suspension of passenger transport did not stop importation [5, 56]. Evidence from the coronavirus disease 2019 pandemic suggests that travel measures have limited and mostly early effects [73]. The East African Community health ministers agreed in June 2026 to harmonise screening at borders [74], and the bilateral memorandum launched later that month, with joint treatment and laboratory capacity at the border, is the kind of instrument whose absence this review identifies; it came after the onset and has not been evaluated [51]. Countermeasure access shows a similar pattern. Neither country had a Bundibugyo-specific provision, but Uganda had a documented route from regulatory and ethics approval to the use of investigational products, used in 2022 and 2025 and again in 2026 through compassionate use of remdesivir [46, 52, 53, 58]. The DRC's standing instruments contain no such route, and the first treatment trial there began enrolling on 2 July [75].

The findings fit the wider literature in two respects. Reviews of epidemic responses in sub-Saharan Africa report poor coordination, weak accountability and weak information systems as constraints [76, 77]; in the DRC the 2023 JEE recorded weak IHR coordination, and surveillance was among the dimensions in which the Congolese instruments were weakest. At the same time, capacity scores have predicted outbreak performance poorly elsewhere [78], and self-assessed scores tend to exceed external ones [79]. Agreement between the JEE, SPAR and the documentary charting in this review suggests that the difference in documented preparedness between the two countries does not rest on any single source, but it does not show that this difference explains the different courses of the outbreak. Insecurity, population movement, the location of the first cases and the extent of transmission before detection differed between the two countries [1, 5, 56], and none of them is measured here.

For the DRC, the priorities indicated are a legal basis for the emergency operations centre and for quarantine, surveillance provisions with timeliness standards and continuity in insecure areas, minimum isolation capacity, a link between national surveillance and the humanitarian health services that reach displacement sites, and publication of its national action plan for health security. For both countries, they are a standing cross-border protocol with notification timelines and criteria for movement restrictions, which could build on the memorandum of June 2026, and a national pathway for the regulatory and ethics review of investigational products that names Bundibugyo virus. For Uganda, they are publication of its current action plan for health security and refugee response plan, and infection prevention and control, in which its JEE score was low and the outbreak exposed a gap.

The review has strengths. Every included source was verified against its issuing body's record, and the copy of each instrument that was read is identified. Every charted provision is quoted with its page in Additional file 4, and the descriptors follow a fixed rule that a reader can reapply. The documentary findings were also set against external evaluation data and against the documented course of the outbreak.

It also has limitations. Three national instruments had no public text, and predecessors could be charted for only two of them; five charted instruments were read from uncertified copies hosted outside the issuing body's website. Uganda's Public Health Act and Constitution could be read only in part, so a provision outside the sections read may have been missed. Uganda had more charted instruments than the DRC, which raises its coverage and could move its modal specificity in either direction. The search was limited to publicly available documents in English and French, so unpublished instruments, internal after-action reviews and subnational plans may contain provisions that the review could not chart. The protocol was agreed by the review team before the first search but was not registered. The specificity ratings and risk descriptors rest on the reviewers' reading of policy text; the rule is fixed and every rating carries a recorded reason, but another team could rate individual provisions differently, and resolving ties in specificity downwards would change three descriptors, although not the direction of the comparison. The descriptors summarise documentary judgements and are not measurements, and the review does not assess whether provisions were carried out. The JEE scores describe capacity at one point in 2023, and SPAR scores are self-reported. The outbreak data are provisional reports from an active outbreak, and the risk descriptors reflect the documentation to 20 June 2026, not the later course of the outbreak in the DRC. The comparison concerns two countries and one outbreak, and its findings may not apply to other settings. Above all, a review of documents cannot establish that any provision caused any outcome.

## Conclusions

The national preparedness instruments of Uganda that were in force when the 2026 Bundibugyo virus disease outbreak began were more complete and more specific than those of the DRC, and Uganda's externally assessed capacity was higher on most JEE indicators. The DRC wrote a specific response plan within two weeks of the declaration, which suggests that the DRC's gap lay in standing capacity and in the instruments that sustain it rather than in planning once an outbreak had begun; no Ugandan response plan for the 2026 outbreak was available for comparison. These findings are consistent with preparedness architecture contributing to the different courses of the outbreak, but a documentary review cannot establish causation. The priorities indicated are surveillance, isolation capacity and a legal basis for emergency operations in the DRC; standing cross-border arrangements and national pathways for investigational Bundibugyo products in both countries; and publication of Uganda's current health security and refugee response plans. Evaluations of how these instruments work in practice, particularly in displacement settings and at the border, are needed.

## Supplementary Information

Additional file 1. Completed PRISMA-ScR checklist, with the location in the manuscript of each reporting item.

Additional file 2. Supplementary methods: periods and language of the review, derivation of the charting framework, verification of sources, charting procedures and quantitative summaries.

Additional file 3. Search strategy by information source, with the screening yield of the June 2026 search and the retrieval of instruments from the issuing bodies at revision.

Additional file 4. Charting workbook: characteristics and verification status of every source, the copy of each instrument read, every charted provision with its quotation and page, component coverage, the descriptors calculated by formula, the JEE and SPAR scores, the outbreak evidence and the verification log.

Additional file 5. The eleven-dimension charting framework, with the component requirements of each dimension and the instrument from which each was drawn.

Additional file 6. Operational definitions and decision rules for the adequacy and outbreak-response risk descriptors, with the documentary basis of every descriptor reported in Tables 2 and 3.

## Data Availability

No datasets were generated or analysed during the current study.

## Abbreviations

Africa CDC: Africa Centres for Disease Control and Prevention
COUSP: Centre des Opérations d'Urgences de Santé Publique (public health emergency operations centre of the DRC)
DRC: Democratic Republic of the Congo
EOC: Emergency operations centre
IHR: International Health Regulations (2005)
INSP: Institut National de Santé Publique (National Institute of Public Health of the DRC)
JBI: Joanna Briggs Institute
JEE: Joint External Evaluation
PRISMA: Preferred Reporting Items for Systematic reviews and Meta-Analyses
PRISMA-ScR: PRISMA extension for Scoping Reviews
SPAR: States Parties Self-Assessment Annual Reporting
UNHCR: United Nations High Commissioner for Refugees
WHO: World Health Organization
