# Supplementary material for: National Ebola preparedness policy architecture and health system readiness in the Democratic Republic of the Congo and Uganda: a scoping review anchored in the 2026 Bundibugyo outbreak
Source: Infect Dis Poverty. 2026 Nov 5;15:114. doi: 10.1186/s40249-026-01521-x (PMC13737950; doi:10.1186/s40249-026-01521-x)
Supplement: Supplementary file 1 [file 40249_2026_1521_MOESM1_ESM.docx]

**Additional file 1.** Completed PRISMA-ScR checklist (Preferred Reporting Items for Systematic reviews and Meta-Analyses extension for Scoping Reviews).

| **Section** | **No.** | PRISMA-ScR checklist item | Reported in |
| --- | --- | --- | --- |
| Title | 1 | Identify the report as a scoping review. | Title page |
| Abstract | 2 | Provide a structured summary (background, objectives, eligibility, sources, charting methods, results, conclusions). | Abstract |
| Introduction | 3 | Describe the rationale for the review in the context of what is already known. | Background |
|  | 4 | Provide an explicit statement of the questions and objectives. | Background (aim); Methods, review questions |
| Methods | 5 | Indicate whether a protocol exists and whether/where it can be accessed; registration. | Methods, protocol and reporting framework |
|  | 6 | Specify characteristics of the sources of evidence used as eligibility criteria. | Methods, eligibility criteria |
|  | 7 | Describe all information sources and the date last searched. | Methods, information sources and search; Additional file 3 |
|  | 8 | Present the full electronic search strategy for at least one source. | Additional file 3 |
|  | 9 | State the process for selecting sources of evidence. | Methods, selection of sources of evidence and verification of sources at revision; Figure 2 |
|  | 10 | Describe the methods of charting data. | Methods, data charting; Additional files 2 and 4 |
|  | 11 | List and define all variables for which data were sought. | Methods, data charting; Additional file 5 (52 component requirements) |
|  | 12 | If done, provide a rationale for conducting a critical appraisal of sources; describe the methods used. | Methods, source credibility; Additional file 4 |
|  | 13 | Describe the methods of handling and summarising the charted data. | Methods, synthesis and analysis, and gap analysis and outbreak-response risk; Additional file 6 |
| Results | 14 | Give numbers of sources screened, assessed, and included, with reasons for exclusions (flow diagram). | Results, selection of sources of evidence; Figure 2 |
|  | 15 | For each source, present characteristics for which data were charted. | Results, characteristics of sources of evidence; Table 1; Additional file 4 |
|  | 16 | If done, present data on critical appraisal of sources. | Table 1; Additional file 4 (source credibility) |
|  | 17 | For each included source, present the relevant data charted. | Results; Additional file 4 |
|  | 18 | Summarise and/or present the charting results as they relate to the review objectives. | Results; Tables 2 and 3; Figures 3 to 5 |
| Discussion | 19 | Summarise the main results, linking to the review objectives; consider their relevance. | Discussion, first paragraph |
|  | 20 | Discuss the limitations of the scoping review process. | Discussion, final paragraph |
|  | 21 | Provide a general interpretation of the results and their implications; note next steps. | Discussion; Conclusions |
| Funding | 22 | Describe sources of funding and the role of funders for the review. | Declarations, funding |

PRISMA-ScR Preferred Reporting Items for Systematic reviews and Meta-Analyses extension for Scoping Reviews.
