# Supplementary material for: National Ebola preparedness policy architecture and health system readiness in the Democratic Republic of the Congo and Uganda: a scoping review anchored in the 2026 Bundibugyo outbreak
Source: Infect Dis Poverty. 2026 Nov 5;15:114. doi: 10.1186/s40249-026-01521-x (PMC13737950; doi:10.1186/s40249-026-01521-x)
Supplement: Supplementary file 2 [file 40249_2026_1521_MOESM2_ESM.docx]

# Additional file 2

**Title:** Supplementary methods: periods and language of the review, derivation of the charting framework, verification of sources, charting procedures and quantitative summaries.

**Associated manuscript:** National Ebola preparedness policy architecture and health system readiness in the Democratic Republic of the Congo and Uganda: a scoping review anchored in the 2026 Bundibugyo outbreak.

## S1. Contextual period and search period

The contextual period of the review runs from 2007, when Bundibugyo virus was first identified in Bundibugyo district, Uganda [8], to the 2026 outbreak. The first search was restricted to documents published between January 2018 and June 2026, because the instruments in force in 2026, and the evaluations that assess them, date from the period after the 2018 to 2020 outbreak in North Kivu and Ituri. Instruments issued before 2018 that remained in force, such as Uganda's Public Health Act and Constitution, were identified through reference lists, through their incorporation by reference into later instruments and through the targeted retrieval at revision. The literature on the identification of the virus in 2007 and its reappearance in 2012 is cited in the Background as scientific context and was not charted.

## S2. Language of sources

Sources in English and French were eligible. The French sources were the Congolese national instruments, the Congolese public health law and its 2023 amendment, the Joint External Evaluation (JEE) reports of the Democratic Republic of the Congo (DRC) and the Congolese national situation report of 11 June 2026. They were charted from the French text, and the quotations in the charting workbook (Additional file 4) are given in French so that a reader can check the reading on which each charted judgement rests. Where a French term has an administrative meaning with no exact English equivalent, such as zone de santé (health zone) or the Centre des Opérations d'Urgences de Santé Publique (COUSP), the French term is kept alongside the English rendering.

## S3. Derivation of the charting framework

The ten substantive dimensions group the component requirements of the World Health Organization (WHO) consolidated Ebola virus disease preparedness checklist [30] and of the third edition of the JEE tool [31] by the object of policy they address. The checklist sets out eleven components (coordination, rapid response team, public awareness and community engagement, infection prevention and control, case management, epidemiological surveillance, contact tracing, laboratory, capacities at points of entry, budget and logistics), and the JEE tool sets out nineteen technical areas. A group was retained as a dimension where both instruments contained a corresponding requirement. Two dimensions are exceptions. Inclusion of displaced populations is not a separate component of either instrument, and its components were drawn from the case for including refugees in national public health systems and from work on public health information in crises [32, 33]. Operational specificity is a property of policy text rather than a substantive requirement. The areas of the joint Africa Centres for Disease Control and Prevention (Africa CDC) and WHO continental preparedness and response plan for the 2026 outbreak (coordination, surveillance, laboratory testing, infection prevention and control, clinical care, community engagement, research, logistics, cross-border collaboration and the protection of vulnerable populations) each correspond to one or more of the dimensions [34]. The components and their provenance are listed in Additional file 5.

## S4. Alignment with WHO and Africa CDC standards

The alignment dimension asks three questions of each national instrument: whether it refers to the relevant WHO or Africa CDC standard; whether it maps its own provisions against that standard, benchmark by benchmark, in a form that shows which requirements are met; and whether it names an institution and a review cycle responsible for closing the gaps that such a mapping identifies. The WHO standards are those of the International Health Regulations (IHR, 2005) as operationalised in the JEE tool and in States Parties Self-Assessment Annual Reporting (SPAR) [31, 35]; the Africa CDC standards are those reflected in the joint continental plan [34]. The assessment is documentary. It establishes whether an instrument is written so as to be accountable to these standards, not whether the standards are met in practice.

## S5. Verification of sources and charting at revision

Every source included in the first version of the review was checked against the issuing body's own publication record, and every journal citation against PubMed. A source was recorded as traced when a document by the stated issuing body with the stated or a corrected title, date or edition could be found; as corrected when the details of a traced document had been cited wrongly; and as not traced when no such document could be found. The outcome for each of the 58 sources, and for the five methodological references of the first version, is given in the verification log of Additional file 4. Where a national instrument cited in the first version could not be traced, it was not replaced by assumption: the instrument of the same type in force on 15 May 2026 was retrieved and charted in its place, or its most recent public predecessor where the instrument in force had no public text.

Each national instrument was read from the issuing body's copy or, where that copy could not be found or opened, from the copy identified in Additional file 4; image-only pages were read from the page image. Most were read in full. The exceptions, recorded instrument by instrument in Additional file 4, are the costing slide and some annex tables of the DRC development plan for 2024 to 2033, which were illegible in the only copy available, the image-only project profiles in an annex of Uganda's Ministry of Health strategic plan II, Uganda's Public Health Act, of which the table of contents and the sections bearing on the components were read, and Uganda's Constitution, of which the National Objectives, Articles 1 to 103 and Article 110 were read. For each of the 52 component requirements, the instrument was coded yes when it contained a provision on the subject of the component, even a generic one; no when no such provision was found; and not determinable when the part of the instrument most likely to contain it could not be read, which occurred once, for the Ugandan Constitution. Each provision coded yes was given a specificity rating with a one-line reason, a summary, a verbatim quotation of about 40 words or fewer in the original language and its printed page. Descriptions of past achievements, situation analyses and statements of context were not counted as provisions. Codings that turned on a broad reading of related text are flagged in the workbook.

## S6. Descriptor rule and treatment of ties

The descriptor for each country and dimension combines coverage of the component requirements with the modal specificity of the provisions addressing them, as set out in Additional file 6, Part A. A tie between specificity levels was resolved upwards. Ties arose in three readings: inclusion of displaced populations and health workforce readiness for the DRC, and alignment with standards for Uganda. Resolving them downwards would make the first two inadequate and the third partially adequate, and Uganda would still be rated higher than the DRC in six of the eleven dimensions.

## S7. Outbreak documentation and risk descriptors

The outbreak documentation was used to describe the context of the review and to assign risk descriptors, not as primary findings. Figures were taken from the source dated closest to the data cut-off (11 June 2026 for case counts and 20 June 2026 for the risk descriptors). Where two sources gave different figures for the same date, both are reported, as for contact follow-up in Ituri. The later course of the outbreak, to 23 September 2026 in the DRC and to its end on 27 August 2026 in Uganda, is reported separately. Each risk descriptor was assigned by reading what the documentation records about the operational consequence of the gap in question, using the definitions in Additional file 6, Part C. The documentary basis for each descriptor is in Additional file 6, Part D, and in Additional file 4.

## S8. Quantitative summaries

JEE indicator scores for the October 2023 missions were read from the mission reports, once from the summary score table and once from the chapter headings; the two readings agreed for all 56 indicators in both countries [48, 49]. The paired comparison treats the 56 indicators as matched pairs. The mean difference is reported with a *t*-based 95% confidence interval and the Wilcoxon signed-rank test, used to describe the consistency of the difference across indicators rather than to generalise to a wider population. SPAR average capacity scores were taken from the WHO Global Health Observatory, using indicator SDGIHR2018 for 2018 to 2020 and SDGIHR2021 from 2021, when the tool changed from 13 to 15 capacities [35]. The paired test was run in Python 3.11 (Python Software Foundation, Wilmington, Delaware, USA; https://www.python.org) with the SciPy library. Counts, percentages, descriptors and the confidence interval were calculated in Python and are recalculated by formula in Additional file 4.

Africa CDC Africa Centres for Disease Control and Prevention, COUSP Centre des Opérations d'Urgences de Santé Publique, DRC Democratic Republic of the Congo, IHR International Health Regulations (2005), JEE Joint External Evaluation, SPAR States Parties Self-Assessment Annual Reporting, WHO World Health Organization. Reference numbers in this file correspond to the reference list of the manuscript.
