# Supplementary material for: National Ebola preparedness policy architecture and health system readiness in the Democratic Republic of the Congo and Uganda: a scoping review anchored in the 2026 Bundibugyo outbreak
Source: Infect Dis Poverty. 2026 Nov 5;15:114. doi: 10.1186/s40249-026-01521-x (PMC13737950; doi:10.1186/s40249-026-01521-x)
Supplement: Supplementary file 3 [file 40249_2026_1521_MOESM3_ESM.docx]

**Additional file 3.** Search strategy by information source. In the June 2026 search, concept blocks were combined with the Boolean operator AND, and terms within each block were combined with OR and adapted to the syntax of each source. As recorded by the review team, the June search identified 659 records; after removal of 78 duplicates, 581 were screened, 83 were sought in full, 79 were assessed and 53 were included. The five documents added by the targeted search of September 2026 were included in the first version of the review. At revision, every source was verified and the national instruments in force at the onset of the outbreak were sought from the issuing bodies (last two rows); the resulting selection is shown in Figure 2 of the manuscript and listed in Additional file 4.

| Source | Type | Search approach and illustrative terms | **Date** |
| --- | --- | --- | --- |
| PubMed (MEDLINE) | Bibliographic database | Three concept blocks combined with AND. Block 1 (pathogen): "Ebola"[tiab] OR "Bundibugyo"[tiab] OR ebolavirus[tiab]. Block 2 (concept): preparedness OR "health system readiness" OR "epidemic governance" OR "International Health Regulations" OR surveillance OR "health security". Block 3 (context): "Democratic Republic of the Congo" OR DRC OR Congo OR Uganda. Limits: 2018/01/01 to 2026/06/30; English, French. | June 2026 |
| Global Health (CABI) | Bibliographic database | Same three concept blocks adapted to CABI thesaurus terms and field tags; 2018 to 2026; English, French. | June 2026 |
| WHO IRIS | Grey-literature repository | Repository search on Ebola AND (preparedness OR surveillance OR "health regulations") filtered to DRC and Uganda country tags; JEE and situation-report collections browsed. | June 2026 |
| Africa CDC publications | Grey-literature repository | Browsed health-security and outbreak-update collections for Bundibugyo, DRC and Uganda documents. | June 2026 |
| EAC Secretariat archive | Grey-literature repository | Searched health-security and cross-border epidemic-governance documents. | June 2026 |
| UNHCR data and documentation portal | Grey-literature repository | Searched health-policy and DRC-situation collections for displacement and health-inclusion documents. | June 2026 |
| DRC and Uganda health ministry sites | Official government sites | Retrieved national health sector plans and national Ebola preparedness and response frameworks. | June 2026 |
| Reference lists of included sources | Hand-searching | Backward citation searching of all included sources. | June 2026 |
| Targeted search, September 2026 | Named instruments | Ugandan public health legislation, the Ugandan national action plan for health security and UNHCR operational data for Uganda, searched by title on the issuing bodies' websites and the Uganda Legal Information Institute. | September 2026 |
| Issuing bodies at revision | Official repositories | Instruments in force on 15 May 2026 sought from the INSP (https://insp.cd), the Uganda Ministry of Health and its library, the WHO Strategic Partnership for Health Security portal, WHO IRIS, WHO Disease Outbreak News, the WHO Regional Office for Africa, Africa CDC, the UNHCR data portal and the Uganda Legal Information Institute; JEE mission reports; SPAR data from the WHO Global Health Observatory; outbreak documentation to 1 October 2026. | 1 October 2026 |
| PubMed verification at revision | Bibliographic database | Every journal citation of the first version checked by title, first author, journal and year, and replacement literature identified by title and keyword searches. | 1 October 2026 |

Africa CDC Africa Centres for Disease Control and Prevention, CABI Centre for Agriculture and Bioscience International, DRC Democratic Republic of the Congo, EAC East African Community, INSP Institut National de Santé Publique, IRIS Institutional Repository for Information Sharing, JEE Joint External Evaluation, SPAR States Parties Self-Assessment Annual Reporting, UNHCR United Nations High Commissioner for Refugees, WHO World Health Organization.
