# Supplementary material for: National Ebola preparedness policy architecture and health system readiness in the Democratic Republic of the Congo and Uganda: a scoping review anchored in the 2026 Bundibugyo outbreak
Source: Infect Dis Poverty. 2026 Nov 5;15:114. doi: 10.1186/s40249-026-01521-x (PMC13737950; doi:10.1186/s40249-026-01521-x)
Supplement: Supplementary file 4 [file 40249_2026_1521_MOESM5_ESM.docx]

# Additional file 5

**Title:** The eleven-dimension charting framework: component requirements charted under each dimension and the source instrument from which each component was drawn.

**Associated manuscript:** National Ebola preparedness policy architecture and health system readiness in the Democratic Republic of the Congo and Uganda: a scoping review anchored in the 2026 Bundibugyo outbreak.

The eleven analytical dimensions used to chart the national instruments were derived from the World Health Organization (WHO) consolidated Ebola virus disease preparedness checklist [30] and the technical areas of the third edition of the Joint External Evaluation (JEE) tool [31]. The components of the displaced-population dimension were drawn from published work on the inclusion of refugees in national public health systems and on public health information in crisis-affected populations [32, 33], and operational specificity is a property of policy text added by the review team. Each area of the joint Africa Centres for Disease Control and Prevention (Africa CDC) and WHO continental preparedness and response plan for the 2026 outbreak corresponds to one or more of the dimensions [34]. Component requirements were grouped by the object of policy they address and retained as a dimension where both WHO instruments contained a corresponding requirement; the two exceptions are marked in the table. Charting was carried out at the level of the component requirement: each national instrument was searched for a provision addressing each component, and the provision found, or its absence, was recorded in the charting workbook (Additional file 4). Every instrument cited in the provenance column can be traced to the issuing body's own publication record.

| **Analytical dimension** | **Component requirements charted under the dimension** | **Source instrument and locus** |
| --- | --- | --- |
| 1. Preparedness governance architecture | National coordination body with named chair and membership; command and control and escalation arrangements; replication of the coordination structure at subnational level; statutory basis for declaring and managing an epidemic emergency; activation criteria and thresholds. | WHO checklist: coordination [30]. JEE tool: P1 legal instruments; P3 IHR coordination, national IHR focal point functions and advocacy; R1 health emergency management [31]. |
| 2. Surveillance system design and coverage provisions | Community-based event detection; health facility indicator-based surveillance; laboratory confirmation pathway; electronic reporting with a defined timeliness standard; screening at points of entry; case definitions in operational use; provisions for surveillance continuity in insecure or inaccessible areas. | WHO checklist: epidemiological surveillance; contact tracing; laboratory; capacities at points of entry [30]. JEE tool: D1 national laboratory system; D2 surveillance; PoE points of entry [31]. |
| 3. Inclusion of forcibly displaced populations in surveillance and response (exception 1) | Registration of displaced populations within surveillance denominators; deployment of community health workers in camps and settlements; a data-sharing arrangement between the national surveillance system and humanitarian health information systems; a named coordination counterpart for refugee and internally displaced person affairs; provision for service continuity where humanitarian actors deliver care. | Not a separate component of the WHO checklist or a technical area of the JEE tool, which is why the dimension is recorded as an exception. Components drawn from the case for including refugees and migrants in national public health systems and from work on public health information in crisis-affected populations [32, 33]. |
| 4. Isolation and treatment capacity planning | Designated treatment facilities named in the framework; minimum bed provision or bed-to-population ratio; surge triggers and thresholds; pre-positioned materials for rapid establishment of treatment units; patient referral and transport arrangements; infection prevention and control standards for isolation areas. | WHO checklist: infection prevention and control; case management [30]. JEE tool: R1 health emergency management; R3 health services provision; R4 infection prevention and control [31]. |
| 5. Health workforce readiness provisions | Cadres to be trained and the content of their training; training frequency and competency assessment; a rapid response team roster with activation criteria; personal protective equipment provision and stockpile requirements; deployment, supervision and compensation arrangements for responders. | WHO checklist: rapid response team [30]. JEE tool: D3 human resources; R1 health emergency management [31]. |
| 6. Cross-border epidemic governance mechanisms | Bilateral notification obligations with stated timelines; joint surveillance or joint investigation arrangements; border health post protocols; criteria governing movement restriction and its humanitarian mitigation; regional reporting obligations to the East African Community and to Africa CDC. | WHO checklist: capacities at points of entry [30]. JEE tool: PoE points of entry; P3 IHR coordination, national IHR focal point functions and advocacy [31]. |
| 7. Community engagement and risk communication provisions | A named unit responsible for risk communication; a communication plan differentiated by audience and setting; a community feedback mechanism; provisions for managing rumour and misinformation; engagement provisions specific to displacement settings and to areas of insecurity; safe and dignified burial provisions. | WHO checklist: public awareness and community engagement; case management, including safe burial [30]. JEE tool: R5 risk communication and community engagement [31]. |
| 8. Medical countermeasure access provisions | Strain-specific product provisions; a regulatory pathway for investigational products; an ethics pathway, including provision for the monitored emergency use of unregistered and investigational interventions; stockpiling and allocation arrangements; research and development coordination with regional and international partners. | WHO checklist: budget; logistics [30]. JEE tool: R1 health emergency management; P8 immunization [31]. |
| 9. Accountability and monitoring frameworks for preparedness | Defined preparedness indicators; a periodic review cycle with a named owner; a requirement for after-action review; a mechanism carrying Joint External Evaluation and after-action findings into the next framework revision; provision for independent or external review. | WHO checklist: coordination [30]. JEE tool: P2 financing; P3 IHR coordination, national IHR focal point functions and advocacy; R1 health emergency management [31]. |
| 10. Alignment with WHO AFRO and Africa CDC preparedness standards | Reference to the relevant standard within the national framework; mapping of national provisions against the standard, benchmark by benchmark, in a form that shows which requirements are met; a named institution and review cycle responsible for closing the gaps that the mapping identifies. | Cross-cutting. Assessed against the IHR (2005) as operationalised in the JEE tool and in States Parties Self-Assessment Annual Reporting (SPAR) [31, 35], and against the areas of the joint Africa CDC and WHO continental plan [34]. The assessment is documentary and establishes whether an instrument is written so as to be accountable to these standards, not whether the standards are met in practice. |
| 11. Operational specificity depth (exception 2) | For each provision charted under dimensions 1 to 10: whether an institution is named; whether a timeline is stated; whether a resource requirement is quantified; whether an accountable actor is identified for the action specified. | Cross-cutting property of policy text rather than a substantive requirement, added by the review team and recorded as the second exception. The four components reflect the form in which requirements are expressed in the WHO checklist and the JEE tool, each of which specifies actor, action, timing and resource. |

Africa CDC Africa Centres for Disease Control and Prevention, AFRO Regional Office for Africa, IHR International Health Regulations (2005), JEE Joint External Evaluation, PoE points of entry, SPAR States Parties Self-Assessment Annual Reporting, WHO World Health Organization. Codes beginning P, D and R and the code PoE denote technical areas of the JEE tool. Reference numbers in this file correspond to the reference list of the manuscript.
