# Supplementary material for: National Ebola preparedness policy architecture and health system readiness in the Democratic Republic of the Congo and Uganda: a scoping review anchored in the 2026 Bundibugyo outbreak
Source: Infect Dis Poverty. 2026 Nov 5;15:114. doi: 10.1186/s40249-026-01521-x (PMC13737950; doi:10.1186/s40249-026-01521-x)
Supplement: Supplementary file 5 [file 40249_2026_1521_MOESM6_ESM.docx]

# Additional file 6

**Title:** Operational definitions and decision rules for the four-level adequacy descriptors and the four-level outbreak-response risk descriptors, with the documentary basis for every descriptor reported in Tables 2 and 3 of the manuscript.

**Associated manuscript:** National Ebola preparedness policy architecture and health system readiness in the Democratic Republic of the Congo and Uganda: a scoping review anchored in the 2026 Bundibugyo outbreak.

## Part A. The four-level adequacy descriptor

Charting was carried out at the level of the individual provision, and each provision was classified for operational specificity on a three-level scheme: high (concrete actions, named institutions, defined timelines, quantified resource requirements), moderate (general procedural guidance with some institutional attribution but limited quantification) and low (aspirational or generic language without operational detail). The descriptors reported in Table 2 and Figure 3 of the manuscript operate at a different unit of analysis. They apply to a country within a dimension and summarise all provisions charted for that country in that dimension in the national instruments in force at the onset of the outbreak. Two properties of that set were read.

The first is coverage, meaning the proportion of the component requirements listed for the dimension in Additional file 5 that any charted provision addresses. Coverage was recorded as full where every component requirement is addressed by at least one provision, partial where at least one but not all are addressed, and none where no provision addressing the dimension was found in any charted instrument for that country. The second is the modal specificity classification of the provisions that address those components. The rule combining the two was fixed before synthesis and applied uniformly, and is set out in the grid below.

|  | **Modal specificity high** | **Modal specificity moderate** | **Modal specificity low** |
| --- | --- | --- | --- |
| Coverage full | Adequate | Partially adequate | Inadequate |
| Coverage partial | Partially adequate | Partially adequate | Inadequate |
| Coverage none | Absent | Absent | Absent |

Three features of the rule are worth stating explicitly. Coverage of none returns the descriptor absent whatever else the documents contain, because the dimension is then unaddressed rather than weakly addressed. Modal specificity of low returns the descriptor inadequate whether coverage is full or partial, because a provision that states an intention without directing an action does not supply the requirement it names. Full coverage at moderate specificity and partial coverage at high specificity both return partially adequate, because each falls short of adequacy in one respect only, the first in the operational detail of its provisions and the second in the range of requirements they reach.

Where the specificity classifications of the provisions charted in a dimension were evenly split, the higher classification was taken. This arose in three readings: inclusion of displaced populations and health workforce readiness for the Democratic Republic of the Congo (DRC), and alignment with standards for Uganda. Taking the lower classification instead would rate the DRC inadequate on the first two and Uganda partially adequate on the third; Uganda would still be rated higher than the DRC in six of the eleven dimensions. Descriptors were assigned to the national instruments as written. They are not statements about implementation, and they are not measurements.

## Part B. Coverage, modal specificity and documentary basis for each descriptor in Table 2 of the manuscript

The second and fourth columns record, for each country, the coverage reading (with the number of component requirements addressed), the modal specificity reading and the descriptor that the grid in Part A returns from them. The charted provisions supporting each reading, with quotations and page numbers, are given in Additional file 4.

| **Dimension** | **DRC: coverage / modal specificity / descriptor** | **Documentary basis, DRC** | **Uganda: coverage / modal specificity / descriptor** | **Documentary basis, Uganda** |
| --- | --- | --- | --- | --- |
| Preparedness governance | Full (5/5) / moderate / partially adequate | Multi-hazard plan: coordination council for epidemics, emergencies and complex disasters under the Prime Minister, a task force led by the Minister of Health and the COUSP within the INSP; the Minister's declaration activates the COUSP and its incident management system; risk assessment within 120 minutes of an alert [36]. Development plan: establish the national emergency operations centre with a regulatory framework [39]. Public health law as amended: coordination council under the Prime Minister (art. 106) and the INSP charged with applying the IHR (art. 42 sexies), but no power to declare an epidemic or order quarantine [37, 38]. | Full (5/5) / moderate / partially adequate | Public Health Act: the Minister declares epidemic diseases and infected areas and makes rules for their control (ss. 10, 12, 30, 31) [40, 41]; Constitution, article 110, state of emergency [42]; action plan for health security: emergency operations centre and incident management system able to activate within 120 minutes [44]; regional guidelines: modes of operation, activation criteria and notification chain [43]; 2022 Ebola plan: National Task Force, district task forces and an incident management system with nine pillars [46]. |
| Surveillance | Partial (6/7) / low / inadequate | Multi-hazard plan: laboratory network of four pools, 48-hour detection target and community alert definitions, but no Ebola case definition and no timeliness standard for electronic reporting [36]; development plan: generic surveillance actions [39]; public health law: duty to notify epidemic-prone diseases (art. 46) and participation of health staff and communities in surveillance (art. 47) [37]. No provision for surveillance in insecure or inaccessible areas. | Partial (6/7) / moderate / partially adequate | Action plan for health security: surveillance training year by year, seven national laboratories and specimen transport from at least 80% of districts [44]; strategic plan II: detection within 72 hours, confirmation within 7 days and functional points of entry rising from 8 to 17 [45]; regional guidelines: daily reporting and the 7-1-7 target [43]; 2022 Ebola plan: 24-hour alert desks and a 12-hour laboratory turnaround [46]; refugee response plan: 48-hour detection target [47]. No provision for insecure areas. |
| Inclusion of displaced populations | Partial (2/5) / moderate (tie between low and moderate) / partially adequate | Multi-hazard plan: displacement treated as a hazard led by the Ministry of the Interior, with continuity of primary care for displaced people within 48 hours; no reference to humanitarian providers or to Ebola [36]. Development plan: no provision [39]. | Full (5/5) / high / adequate | Refugee response plan: district and refugee health information systems harmonised under Ministry of Health authority; paid community health workers in refugee-hosting districts; steering committee including the Office of the Prime Minister and UNHCR; settlement facilities moved into the district health system [47]. Strategic plan II: integration of refugee health into government services [45]. |
| Isolation and treatment capacity | Partial (3/6) / low / inadequate | Multi-hazard plan: case-management kits for 500 patients listed as required and none as available; transfer of suspected cases by the border health programme; no named facilities, bed numbers or surge triggers [36]. Development plan: generic isolation strategy [39]. | Partial (4/6) / moderate / partially adequate | Action plan for health security: supplies pre-positioned at regional referral hospitals [44]; strategic plan II: 15 ambulances a year and regional dispatch centres [45]; Public Health Act: conveyance of infectious patients [40]; 2022 Ebola plan: treatment units including Mubende and pre-positioned supplies [46]. No bed numbers or surge triggers. |
| Health workforce readiness | Full (5/5) / moderate (tie between moderate and low) / partially adequate | Multi-hazard plan: monthly roster updates and deployment of rapid response teams within 24 hours; 188 teams and 1000 protective equipment kits listed as required and none as available [36]. Development plan: indicator for health zones with a rapid response team; generic training [39]. | Full (5/5) / moderate / partially adequate | Action plan for health security: cadres and curriculum, twice-yearly exercises for rapid response teams and personnel deployment guidelines [44]; regional guidelines: minimum training package [43]; 2022 Ebola plan: cadre-specific training [46]. No stockpile levels. |
| Cross-border governance | Partial (4/5) / low / inadequate | Multi-hazard plan: Interpol for cross-border information exchange, the migration authority for control of population movement and the border health programme at points of entry [36]; development plan: a permanent IHR collaboration framework with neighbouring countries [39]. No notification timelines or criteria for movement restriction. | Partial (4/5) / low / inadequate | Public Health Act: powers to restrict entry from specified countries and movement out of infected areas, without criteria (ss. 31, 36) [40]; strategic plan II and action plan: points of entry [44, 45]; 2022 Ebola plan: cross-border meetings and communication lines [46]. No notification timelines or regional reporting obligation. |
| Community engagement and risk communication | Full (6/6) / moderate / partially adequate | Multi-hazard plan: national programme responsible for risk communication, audiences differentiated, rumour management, safe and dignified burial within 24 hours in one section and 48 hours in another [36]; development plan: communication plan listed [39]. | Full (6/6) / moderate / partially adequate | Strategic plan II: health promotion department responsible [45]; action plan: national risk communication platform and community feedback [44]; refugee response plan: health promotion in settlements [47]; 2022 Ebola plan: burial teams in each high-risk district and materials in refugee languages [46]; amended Public Health Act: offence of misleading statements on vaccines [41]. |
| Access to medical countermeasures | Partial (1/5) / high / partially adequate | Multi-hazard plan: yearly pre-positioning of response vaccines; the Ebola scenario requires 24,000 vaccine doses and two antibody treatments, products for *Zaire ebolavirus*, listed as required and not available [36]. Public health law: compulsory vaccination may be ordered when an epidemic appears (art. 98), coded no because it names no product, stockpile or pathway [37]. No regulatory or ethics pathway and no Bundibugyo-specific provision. | Partial (4/5) / moderate / partially adequate | 2022 Ebola plan: available vaccine noted as specific to *Zaire ebolavirus*; approvals from an institutional review board, the Uganda National Council for Science and Technology and the National Drug Authority under a compassionate approach; allocation by ring vaccination [46]; action plan: national framework for medical countermeasures [44]. No Bundibugyo-specific provision. |
| Accountability and monitoring | Partial (4/5) / moderate / partially adequate | Multi-hazard plan: indicators without baselines or targets, annual internal evaluation and an external mid-term evaluation [36]; development plan: four emergency indicators with baselines and annual targets and a review cycle [39]. No after-action review requirement. | Full (5/5) / high / adequate | Action plan for health security: after-action reviews for every activation of the emergency operations centre and an annual review using the WHO benchmarks, SPAR, simulation exercises and after-action reviews [44]; strategic plan II: quarterly and annual reviews [45]; refugee response plan and 2022 Ebola plan: indicators with baselines or targets [46, 47]. |
| Alignment with WHO and Africa CDC standards | Partial (2/3) / moderate / partially adequate | Multi-hazard plan: refers to the IHR and to the 2018 and 2023 JEEs and states that it responds to JEE recommendations, without itemising gaps; Africa CDC not mentioned [36]; development plan: refers to IHR performance by technical area [39]; public health law: INSP charged with the IHR [38]. No benchmark-by-benchmark mapping. | Full (3/3) / high (tie between high and moderate) / adequate | Action plan for health security: reports the 2017 JEE score for each indicator with gaps and objectives; implementation tracked by the Office of the Prime Minister with six-monthly checks until the next JEE [44]; strategic plan II and regional guidelines refer to the IHR [43, 45]. |
| Operational specificity | Not applicable / low / low | Of 66 provisions in the DRC instruments in force at the onset, 10 were rated high, 26 moderate and 30 low [36–39]. The response plan issued in May 2026, charted separately, had 21 high, 11 moderate and 5 low among 37 provisions [55]. | Not applicable / moderate / moderate | Of 142 provisions in the Ugandan instruments, 45 were rated high, 75 moderate and 22 low [40–47]. |

## Part C. The four-level outbreak-response risk descriptor

Risk descriptors were assigned after the policy-gap analysis to eight gap dimensions for both countries. For each, the outbreak documentation up to 20 June 2026 was read and what it records about the operational consequences of the gap was noted. Low denotes that no gap of operational consequence was documented, which is not evidence that none existed.

| **Risk descriptor** | **Operational definition** |
| --- | --- |
| Critical | The 2026 sources document a consequence already impeding case detection, isolation or treatment at scale, or no mitigating provision exists anywhere in the national or regional architecture. |
| Significant | The 2026 sources document an adverse operational consequence confined to part of the affected area, or partly mitigated by provisions elsewhere in the architecture. |
| Moderate | The 2026 sources document a limitation of function without an associated adverse outcome during the outbreak. |
| Low | The 2026 sources document no gap of operational consequence in that dimension. |

## Part D. Documentary basis for each risk descriptor reported in Table 3 of the manuscript

| **Gap dimension** | **DRC** | **Documentary basis, DRC** | **Uganda** | **Documentary basis, Uganda** |
| --- | --- | --- | --- | --- |
| Surveillance coverage | Critical | Surveillance disrupted by insecurity, with suspected cases traced back to March; transmission probably undetected for weeks in newly reporting zones; 28.4% of 6043 contacts seen on 11 June (17.4% in Ituri) in the national report, against 71% to 84% by province in WHO figures for 10 June [4, 5, 56, 62]. | Moderate | First case confirmed on 15 May, the day after the patient died; no community transmission documented; 394 of 820 contacts had completed follow-up by 11 June [1, 5]. |
| Inclusion of displaced populations | Significant | Two Ebola deaths at the Kpangba displacement site near Bunia and a blockade of response teams on 3 June; WHO warned of undetected transmission among mobile and displaced people [5, 20]. | Low | No refugee among the 19 confirmed cases [20]. |
| Isolation and treatment capacity | Critical | 452 isolation beds 69.7% occupied on 11 June, 77.9% in Ituri, where 646 of the 689 cases had occurred; isolation capacity described as inadequate and treatment centres in Ituri as overwhelmed [4, 56, 57]. | Low | Patients isolated in designated centres, including Mulago; no shortfall reported [3, 58]. |
| Health workforce and infection prevention | Critical | 16 health workers infected by 10 June, four of whom died within four days at one hospital; stock-outs of protective equipment and infection prevention materials [5, 56, 63]. | Significant | Four health workers infected while resuscitating a probable case who was embalmed before testing [58]. |
| Cross-border governance | Significant | Cases exported to Uganda, 14 of its 19 cases imported by 11 June; no bilateral protocol before the memorandum of 23 June [5, 51]. | Significant | Passenger transport suspended, contrary to the IHR temporary recommendations; imported cases continued, and movement through informal crossings was expected to continue [5, 56, 59]. |
| Community engagement | Significant | Security incidents and community resistance in Mongbwalu and Rwampara health zones; poor acceptance of safe burial and refusals of post-mortem sampling [4, 56, 61]. | Low | No community resistance reported [5]. |
| Access to medical countermeasures | Critical | No licensed product; treatment and prophylaxis trial protocols still under ethics and regulatory review on 13 June; no national access route in the standing instruments [5, 12]. | Significant | No licensed product; all 18 patients treated at Mulago received remdesivir under a compassionate-use protocol [58]. |
| Coordination and accountability | Moderate | Coordination limitations in remote and insecure areas, although national and provincial coordination had been activated [3, 56]. | Low | No coordination failure reported [3, 5]. |

Africa CDC Africa Centres for Disease Control and Prevention, COUSP Centre des Opérations d'Urgences de Santé Publique, DRC Democratic Republic of the Congo, IHR International Health Regulations (2005), INSP Institut National de Santé Publique, JEE Joint External Evaluation, SPAR States Parties Self-Assessment Annual Reporting, UNHCR United Nations High Commissioner for Refugees, WHO World Health Organization. Reference numbers in this file correspond to the reference list of the manuscript.
